# Mesenteric follicular lymphoma

**DOI:** 10.1002/ccr3.2148

**Published:** 2019-04-12

**Authors:** Yoshito Nishimura, Akira Yamamoto, Masahiro Takahara, Fumio Otsuka

**Affiliations:** ^1^ Department of General Medicine Okayama University Graduate School of Medicine, Dentistry and Pharmaceutical Sciences Okayama Japan

**Keywords:** follicular lymphoma and PET‐CT, mesenteric tumors

## Abstract

When you see a patient with solid mesenteric tumors, malignant lymphoma should be considered as an important differential diagnosis. It is essential to include abdominal CT and ^18^F‐FDG PET/CT examinations in these patients for early diagnosis of malignant lymphoma, giving extra weight on patients' complaints.

## 1 CASE

**Figure 1:**
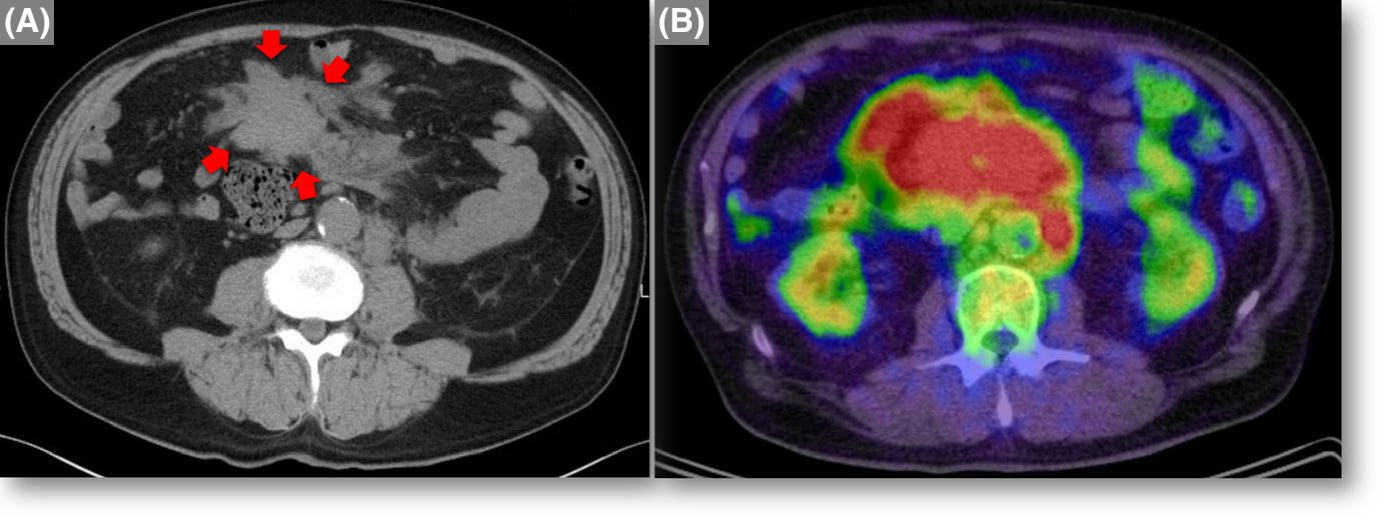
A, Noncontrast CT demonstrated a huge intraperitoneal mass (13 cm diameter) (arrows). B, Fluorine‐18‐fluorodeoxyglucose positron emission tomography‐CT showed a bulky mass with a maximum standardized uptake value of 13.26

A 72‐year‐old man was referred to our hospital for evaluation of a huge mass on CT (Figure 1A, arrows). He presented to the previous hospital with awareness of nontender abdominal mass. He was otherwise asymptomatic. ^18^F‐FDG PET/CT revealed a bulky mesenteric mass (13 cm diameter) with a maximum standardized uptake value of 13.26 (Figure 1B). He underwent laparotomy for excisional biopsy, which were compatible with follicular lymphoma. He was treated with bendamustine plus rituximab. He has been followed up on outpatient basis and has had no recurrent disease to date.

Primary mesenteric tumors are very rare, with incidence of <1 in 200 000, and follicular lymphoma is the most common histological type.^1^ There are various imaging patterns of mesenteric lymphoma at CT; including rounded, enhancing, or homogenous masses. Although the sandwich sign, bulky lymphadenopathy in the mesentery encasing vessels and the bowel, is known to be suggestive of malignant tumors,^2^ its diagnostic performance has been unknown. Because mesenteric lymphoma may be indolent, a focus on patient complaints, such as awareness of mass as this case, is essential for early diagnosis. Among solid mesenteric tumors, malignant lymphoma should be considered a priority.

## CONFLICT OF INTEREST

None declared.

## AUTHOR CONTRIBUTION

YN: involved in writing the manuscript. AY and MT: involved in editing the manuscript. FO: involved in supervision of all procedures.
